# PK/PD Study of Mycophenolate Mofetil in Children With Systemic Lupus Erythematosus to Inform Model-Based Precision Dosing

**DOI:** 10.3389/fphar.2020.605060

**Published:** 2020-12-21

**Authors:** Yewei Chen, Li Sun, Hong Xu, Min Dong, Tomoyuki Mizuno, Alexander A. Vinks, Hermine I. Brunner, Yifan Li, Zhiping Li

**Affiliations:** ^1^ Department of Pharmacy, Children’s Hospital of Fudan University, Shanghai, China; ^2^ Division of Rheumatology, Children’s Hospital of Fudan University, Shanghai, China; ^3^ Division of Clinical Pharmacology, Cincinnati Children’s Hospital Medical Center, Cincinnati, OH, United States; ^4^ Department of Pediatrics, University of Cincinnati, Cincinnati, OH, United States; ^5^ Division of Rheumatology, Cincinnati Children’s Hospital Medical Center, Cincinnati, OH, United States

**Keywords:** mycophenolic acid, pharmacokinetic/pharmacodynamic, children, systemic lupus erythematosus, precision dosing

## Abstract

**Objectives:** To evaluate the mycophenolic acid [MPA, the active form of mycophenolate mofetil (MMF)] pharmacokinetic parameters in relation to clinical response to identify target exposure ranges in pediatric patients with systemic lupus erythematosus (SLE).

**Methods:** This was a retrospective study using pharmacokinetic data collected in 67 pediatric patients aged 4–18 years with SLE. Target MPA exposures for effective inhibition of SLE activity (as measured by SLE disease Activity Index (SLEDAI), active SLE was defined as a SLEDAI score of ≥6, and a controlled disease was defined as a SLEDAI score of ≤4) were assessed by receiver operating characteristic (ROC) curve and logistic regression. Exposure-response models were developed to quantitatively describe the relationship between SLEDAI score and AUC_0–12_ or C_trough_, respectively.

**Results:** The MPA AUC_0-12_ in patients with active SLE was significantly lower than that in patients with inactive SLE. ROC analysis revealed that an AUC_0–12_ threshold of 39 μg h/ml or a C_trough_ of 1.01 μg/ml was associated with the lowest risk of active SLE. Logistic regression analysis revealed that an AUC_0–12_ of less than 34 μg h/ml or a C_trough_ of less than 1.2 μg/ml probably is associated with active SLE. The results of the exposure-response modeling also indicated that an AUC_0-12_ less than 32 μg h/ml or a C_trough_ less than 1.1 μg/ml was associated with suboptimal clinical outcome. An AUC_0-12_ above 50 μg h/ml or a C_trough_ above 1.7 ug/ml was associated with disease control.

**Conclusion:** Both AUC_0–12_ and C_trough_ of MPA are predictive of the likelihood of active SLE in pediatric patients receiving MMF. An individualized dosing regimen of MMF, with a target AUC_0–12_ or C_trough_, should be considered for SLE patients.

## Introduction

Systemic lupus erythematosus (SLE) is a systemic autoimmune disease characterized by the presence of autoantibodies. These antibodies can circulate in the body or form immune complexes that deposit in different organs leading to complement consumption and the production of inflammation cytokines, which leads to local inflammation and organ damage (Tsokos, 2011). Multiple drugs with immunomodulating properties have been used to treat patients with SLE: hydroxychloroquine, corticosteroids, methotrexate, azathioprine and mycophenolate mofetil (MMF) (Tunnicliffe et al., 2015).

MMF is the ester prodrug of mycophenolic acid (MPA). MPA is a potent immunosuppressant that inhibits the *de novo* synthesis pathway of guanosine nucleotides, which triggers a potent cytostatic effect on T and B lymphocytes, thereby inhibiting proliferative response (Allison and Eugui, 2005). With an improved efficacy and safety profile, MPA is being increasingly used in the treatment of SLE. However, a large inter-individual variability in the pharmacokinetics (PK) of MPA has been observed in children with SLE (Zahr et al., 2010; Sherwin et al., 2012). The absorption characteristics of MPA differ on both inter- and intra-patient levels, probably because of the different dosage forms, gastrointestinal tract status and serum albumin level (Tett et al., 2011). The processes of intrinsic clearance and drug transport can also contribute to the variability of MPA PK. MPA is almost entirely metabolized by glucuronidation to its inactive metabolite, MPA-glucuronide (MPAG) (Staatz and Tett, 2014). MPA glucuronides are excreted unchanged in the urine, but also undergo subsequent enterohepatic recirculation (EHR) to convert back to MPA (Villarroel et al., 2009). Individual variation in the rates of metabolism and EHR process lead to significant variability.

To date, several MPA population PK studies have been published (de Winter et al., 2008; Zeng et al., 2010; Sagcal-Gironella et al., 2011; Sherwin et al., 2012; Dong et al., 2014; Woillard et al., 2014; Yoshimura et al., 2018). The majority of studies have focused on the renal transplant population, with only few studies conducted in patients with SLE (Dong et al., 2014). In MMF-treated renal transplant recipients, it has been demonstrated that the area under the zero to 12 h concentration-time curve (AUC_0-12_) is the PK parameter best associated with clinical outcome, with a target range of 30–60 μg h/ml (van Gelder et al., 2006). Therapeutic MPA AUC_0-12_ monitoring has been shown to achieve the target range more quickly than trough monitoring, and significantly reduces the risk of acute rejection in renal transplant patients (Le Meur et al., 2007; Saint-Marcoux et al., 2011). This positive experience in transplantation has led many clinicians to think about the use of MPA therapeutic drug management (TDM) in the context of SLE. Several studies in adult or pediatric patients suffering from autoimmune diseases have shown that TDM using AUC is feasible and that plasma MPA AUCs are likely correlated with disease status (Zahr et al., 2010; Woillard et al., 2014). However, the relationship between MPA exposure and response has not been established in SLE patients. Among the tools to determine the AUC, Bayesian estimators are recognized as the most reliable (Tett et al., 2011). Briefly, a Bayesian estimator allows the calculation of an individual patient's AUC, based on a limited number of blood samples and using a PK model, to provide an individualized dose to achieve the target AUC. In the present study, we 1) evaluated AUC_0-12_ estimation using a Bayesian estimator and a limited sampling strategy in pediatric patients with SLE, 2) explored the relationships between MPA exposure and SLE disease activity in a young patient population.

## Methods

### Patients

Data of patients followed in the Division of Rheumatology of Children's Hospital of Fudan University between December 2015 and December 2017 were included in this study. Patients were enrolled if they fulfilled the following criteria: concentration data for AUC estimation available, diagnosis of SLE according to the American College of Rheumatology classification criteria (Tan et al., 1982; Hochberg, 1997); treatment with MMF at an initial dosage of 20–40 mg/kg/d twice daily, with a maximum of 1.5 g/d, in addition to prednisolone and hydroxychloroquine (HCQ); treatment with MMF (mycophenolate mofetil capsules (Cellcept^®^, Roche Pharmaceuticals, Inc, Palo Alto) or mycophenolate mofetil dispersible tablets (Saikeping, Hangzhou Zhongmei Huadong Pharmaceutical Co., Ltd.)) at a stable dose for at least 10 weeks. Patients were excluded if they were treated with drugs known to effect clinical response, such as cyclosporine, tacrolimus, or methotrexate. Patients' data such as demographics and SLE disease activities were collected at the time of PK evaluation. Only a single time data from each patient were included. The study protocol was approved by the Fudan Children's ethics committee.

### Laboratory Analysis

Blood samples for MPA measurement were drawn as part of standard of care at 30 min before administration of MMF and at 20 min, 1 and 3 h after administration (Prémaud et al., 2005). Plasma concentrations were determined by an enzyme-multiplied immunoassay method with the Viva-E System (ver. 2.014; Siemens Healthcare Diagnostics, Eschborn, Germany). The linear range for the assay was 0.1–15 μg/ml, and the lower limit of quantification was 0.1 μg/ml.

### Pharmacokinetic/Pharmacodynamic Analysis

The MPA AUC_0-12_ was determined using Bayesian estimation with MW/Pharm++ clinical software (Ver. 1.6.1.128; Mediware, Prague, Czech Republic). A published MMF PK model in pediatric patients with SLE was used as the Bayesian prior (Woillard et al., 2014). All MMF doses, times of administration, and serum concentrations were entered into the appropriate sections of the MW/Pharm++ software for the Bayesian estimation. The predictive performance of the model was evaluated as described in Supplementary materials (Supplementary Figures S1, S2; Supplementary Tables S1‒S3). SLE status was assessed using the SLE disease Activity Index (SLEDAI, range: 0–105) (Gladman et al., 2002), active SLE was defined as a SLEDAI score of ≥6, and a controlled disease was defined as a SLEDAI score of ≤4 (Costedoat-Chalumeau et al., 2006).

To evaluate parameters that may have influenced SLE activity on the day of sampling, we conducted a binary logistic regression analysis including statistically (univariate analysis *p*-value < 0.5) and clinically significant parameters. If the *p*-value was less than 0.05 and the odds ratio (OR) was above or below 1.0, then the variables were considered as parameters significantly associated with SLE activity. Parameters significantly associated with AUC_0-12_ or C_trough_ were determined using a multiple linear regression.

A receiver operating characteristic (ROC) curve (a plot of sensitivity vs. 100 minus specificity) was constructed to determine the target MPA AUC and C_trough_ associated with the lowest risk of active SLE, as defined by the SLEDAI score of ≥6.

In addition, logistic regression was used to describe the MPA exposure in association with the likelihood of active SLE. The resulting sigmoid relationships described Eq. 1, 2 provide the proportional probability of active SLE.

$$f\left(LnAUC\right)=\frac{1}{1+e^{\beta 0+\beta 1\cdot LnAUC}}$$ (1)

$$f\left(LnC_{\mathrm{trough}}\right)=\frac{1}{1+e^{\beta 0'+\beta 1'\cdot LnC_{\mathrm{trough}}}}$$ (2)

where *β*_0_, *β*_0_’, *β*_1_ and *β*_1_’ were the parameters to be estimated. The function represents the fraction of the probability of active SLE at a specific MPA exposure (AUC, C_trough_). “Efficacy” means effective control of SLE activity less than 6.

Exposure-response (E-R) models were developed to describe the relationship between AUC, C_trough_ and SLEDAI score, respectively. Sigmoid Emax models were tested to explore the E-R relationships (Eq. 3, 4). A non-parametric bootstrap was conducted, and the median values and the 95 percent confidence intervals were compared with the final parameter estimates (Ette et al., 2003). A visual predictive check using a total of 1,000 simulated datasets was performed (Holford, 2005).

$$E=E0-\frac{E_{\max}\cdot AUC^{\gamma }}{EC_{50}^{\gamma }+AUC^{\gamma }}$$ (3)

$$E=E0-\frac{E_{\max}\cdot C_{\mathrm{trough}}^{\gamma }}{EC_{50}^{\gamma }+C_{\mathrm{trough}}^{\gamma }}$$ (4)

where E represents the pharmacologic effect, E_0_ represents the baseline of SLEDAI, E_max_ represents the maximum SLEDAI, EC_50_ represents the AUC or C_trough_ that produces 50% maximal SLEDAI, γ describes the steepness of the relationship.

## Results

### Patient Characteristics

**FIGURE 1:**
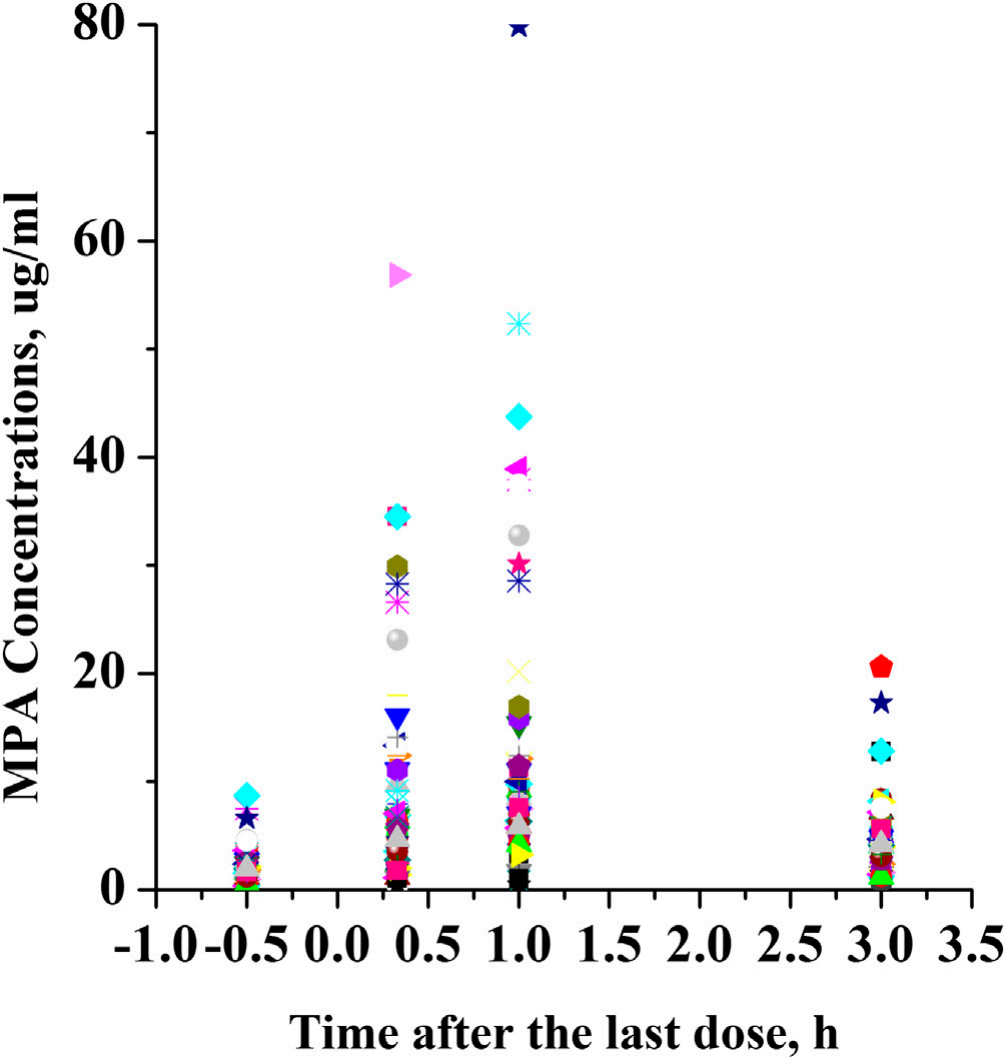
Individual MPA concentration-time profiles.

**FIGURE 2:**
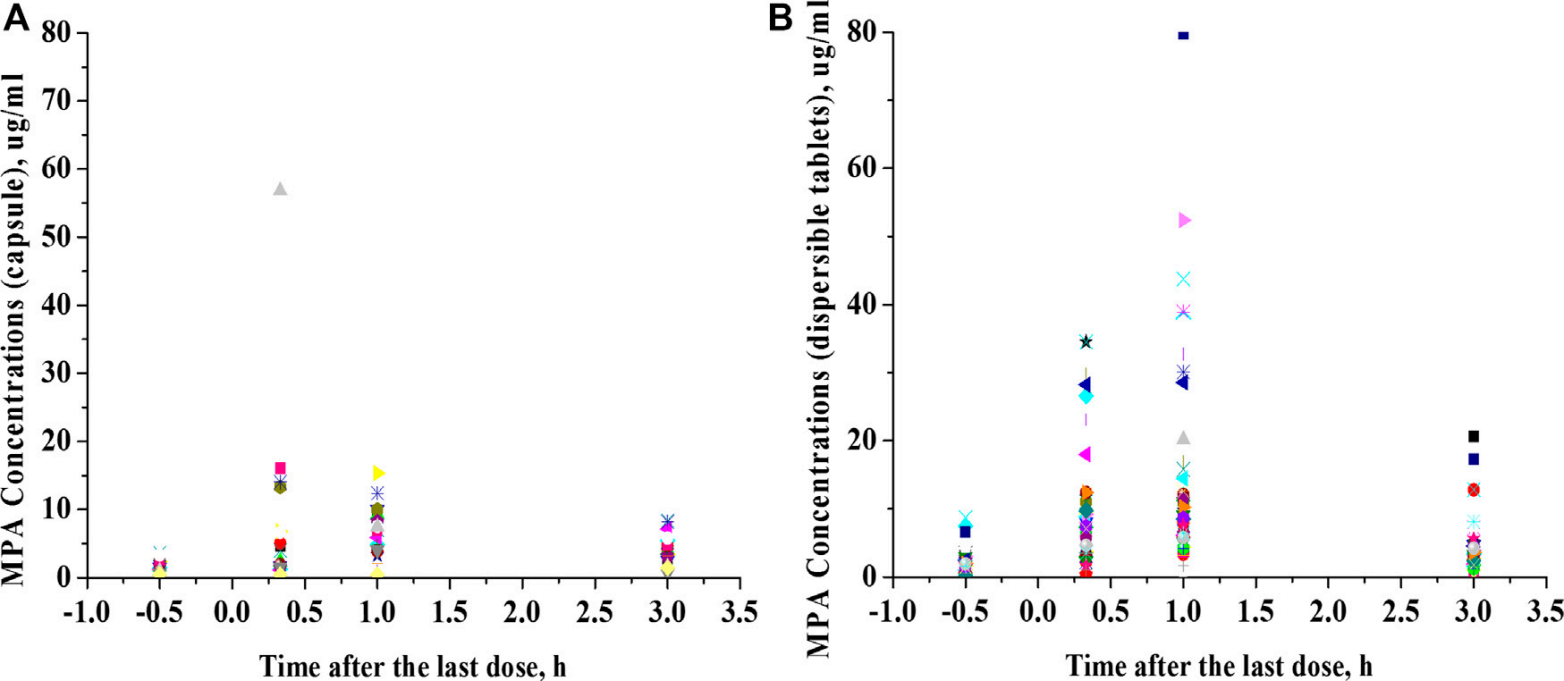
Comparison of PK profiles of different dosage forms. **(A)**, Mycophenolate mofetil capsules. **(B)**, Mycophenolate mofetil dispersible tablets.

**FIGURE 3:**
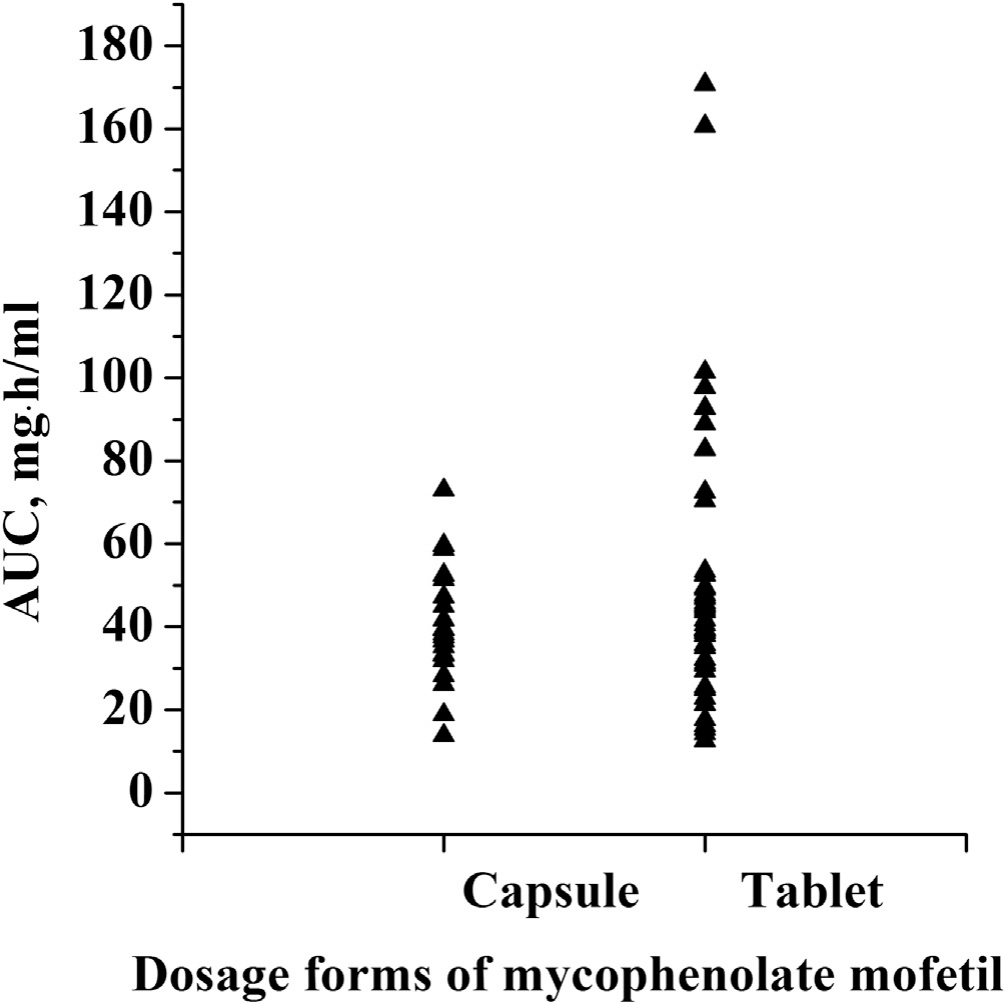
Comparison of AUC results of different dosage forms.

This was a retrospective study that evaluated PK/pharmacodynamic (PD) data of 67 patients. Based on the SLEDAI score, 25 patients had active SLE and 42 patients had inactive SLE on the day of PK sampling (Table 1). In active patients whose diseases were not well controlled during the 10 weeks prior to PK evaluations, dose changes of co-medication could be made based on physicians’ judgment. However, 94% of patients had been on stable dosing of co-medication. Among the patients with active SLE, the mean ± standard deviation (SD) SLEDAI score was 13.4 ± 5.2, and among the patients with inactive SLE, the mean SLEDAI score was 2.6 ± 1.9 (*p* < 0.0001). The active and inactive SLE groups were similar in terms of mean age, mean weight, mean daily dose of MMF, mean daily dose of HCQ and creatinine clearance; but there were significant differences in gender ratio, mean daily dose of steroids and albumin. Individual MPA concentration-time profiles are shown in Figure 1. The comparison of PK profiles and AUC results of different dosage forms is shown in Figures 2, 3, respectively.

**TABLE 1:** Characteristics of the patients with active and inactive SLE, as defined using the SLEDAI score.

	Active SLE (SLEDAI score ≥6) (n = 25)	Inactive SLE (SLEDAI score <6) (n = 42)	*P*
Patients no. (%) (capsule)	10 (15)	11 (16)	
Patients no. (%) (tablet)	15 (22)	31 (46)	
Female no. (%)	24 (96)	32 (76)	0.04
Age, year	12.5 ± 3.1	13.6 ± 2.5	0.30
Weight, kg	45.0 ± 13.3	44.9 ± 9.6	0.93
MMF dosage, mg/kg/day	22.8 ± 5.4	24.0 ± 6.4	0.74
MMF dosage, mg/m^2^/day	744 ± 174	781 ± 195	0.90
Prednisolone dosage, mg/kg/day	0.8 ± 0.5	0.5 ± 0.4	0.016
HCQ, mg/kg/day	4.5 ± 1.8	4.0 ± 0.8	0.28
Creatinine clearance, mL/min/1.73 m^2^	144.0 ± 65.0	152.1 ± 45.2	0.13
Albumin, g/l	33.6 ± 6.6	41.6 ± 3.9	<0.0001
SLEDAI	13.4 ± 5.2	2.6 ± 1.9	<0.0001

a Except where indicated otherwise, values are the mean ± SD. Statistical comparisons were made using the Mann-Whitney *U* test, except for sex which were made using Fisher's exact test; SLEDAI = SLE disease Activity Index; MMF = mycophenolate mofetil; HCQ = hydroxychloroquine.

### Association of MPA Exposure Measures With SLE Activity

**FIGURE 4:**
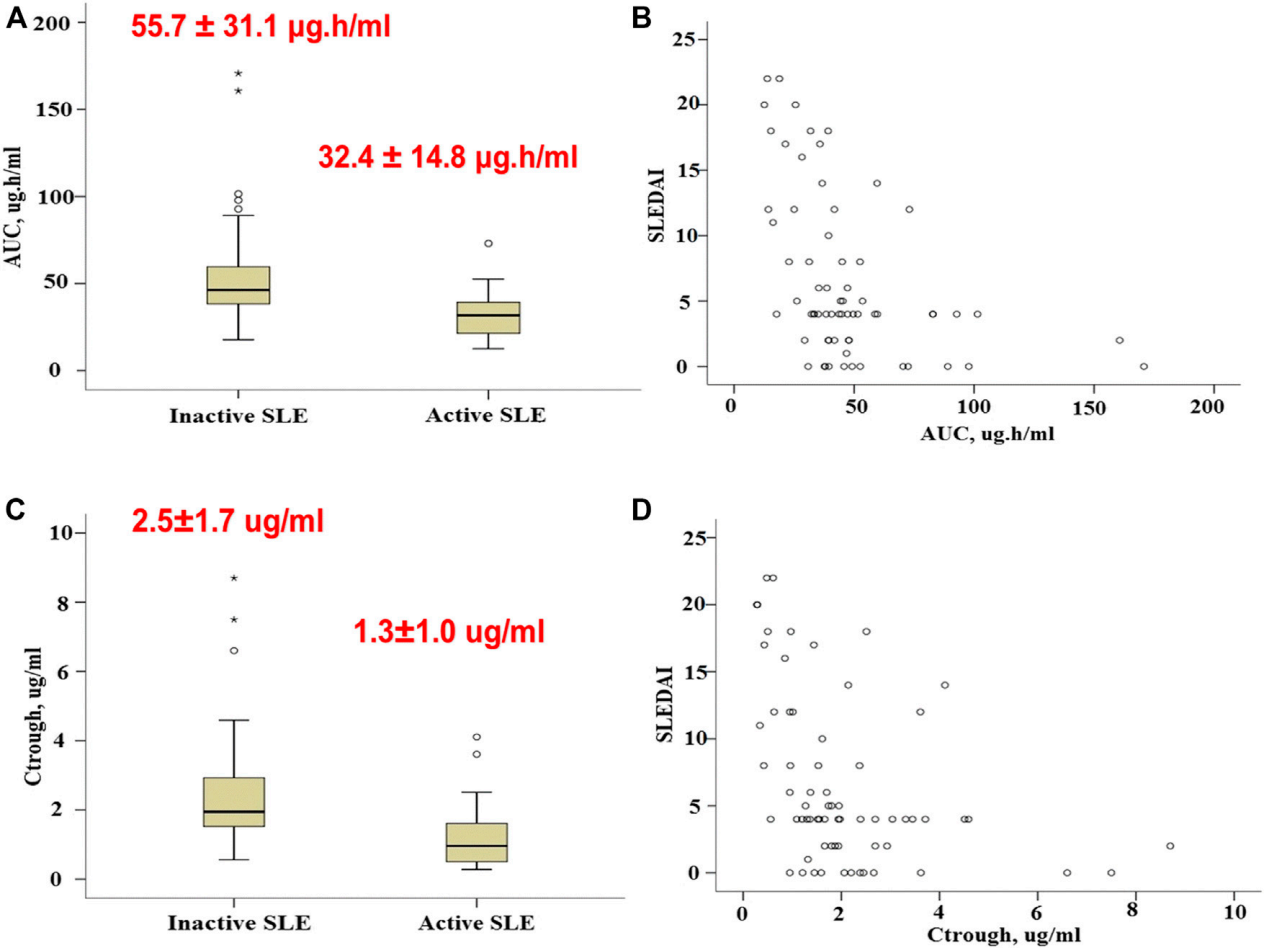
Association of MPA exposure measures with SLE activity. **(A)**, Boxplots of MPA AUC_0–12_ in patients with active and inactive SLE. **(B)**, Association of MPA AUC_0–12_ with the SLEDAI score. **(C)**, Boxplots of C_trough_ in patients with active and inactive SLE. **(D)**, Association of C_trough_ with the SLEDAI score. Each box represents the 25th to 75th percentiles. Lines outside the boxes represent the 10th and the 90th percentiles. Lines inside the boxes represent the median.

MPA AUC_0-12_ displayed wide variability, with a median estimate of 40.6 μg h/ml and a range of 12.6–170.8 μg h/ml. The mean ± SD MPA AUC_0–12_ of the group with active SLE was significantly lower than that of the group with controlled SLE (32.4 ± 14.8 μg h/ml vs. 55.7 ± 31.1 μg h/ml, *p* < 0.0001) (Figure 4A). MPA AUC_0-12_ was associated with the SLEDAI score in a nonlinear fashion (Figure 4B). Similarly, the mean ± SD C_trough_ of the group with active SLE was significantly lower than that of the group with inactive SLE (1.3 ± 1.0 μg/ml vs. 2.5 ± 1.7 μg/ml, *p* < 0.0001) (Figures 4C,D).

### MPA AUC_0–12_ or C_trough_ Is a Major Parameter Influencing SLE Activity

In the simple logistic regression analysis, gender, age, daily dose of HCQ, creatinine clearance and MPA AUC_0–12h_ or C_trough_ were evaluated as independent parameters. MPA AUC_0–12h_ and C_trough_ were identified as the significant independent parameters associated with SLE activity (Table 2).

**TABLE 2:** Binary logistic regression analysis of parameters potentially influencing SLE activity in patients treated with MMF including MPA AUC_0–12_ or C_trough_.

Parameter	OR (95% CI)	*P*
Parameters including MPA AUC_0–12_		
Gender (male)	6.50 (0.63–66.80)	0.12
Age	1.03 (0.79–1.34)	0.84
Daily dose of HCQ	1.19 (0.67–2.12)	0.55
Creatinine clearance	0.997 (0.984–1.011)	0.71
MPA AUC_0–12_	0.93 (0.88–0.98)	0.009
Parameters including MPA C_trough_		
Gender (male)	5.83 (0.61–56.18)	0.13
Age	0.93 (0.74–1.18)	0.57
Daily dose of HCQ	1.28 (0.73–2.25)	0.39
Creatinine clearance	1.000 (0.988–1.012)	0.98
C_trough_	0.43 (0.21–0.87)	0.019

a OR, odds ratio; 95% CI = 95% confidence interval; MPA AUC_0-12_ = mycophenolic acid area under the plasma concentration-time curve from 0 to 12 h.

To assess parameters that may influence MPA AUC_0–12h_ or C_trough_, we constructed a multiple linear regression model including daily dose of MMF, daily dose of steroids, albumin and creatinine clearance. Daily dose of MMF (*p* = 0.005), daily dose of prednisolone (*p* = 0.047), and serum albumin level (*p* = 0.009) were the 3 parameters independently associated with AUC_0–12h_. The linear regression with C_trough_ also indicated that daily dose of MMF (*p* = 0.031), daily dose of prednisolone (*p* = 0.014), and serum albumin level (*p* = 0.041) influenced C_trough_.

**FIGURE 5:**
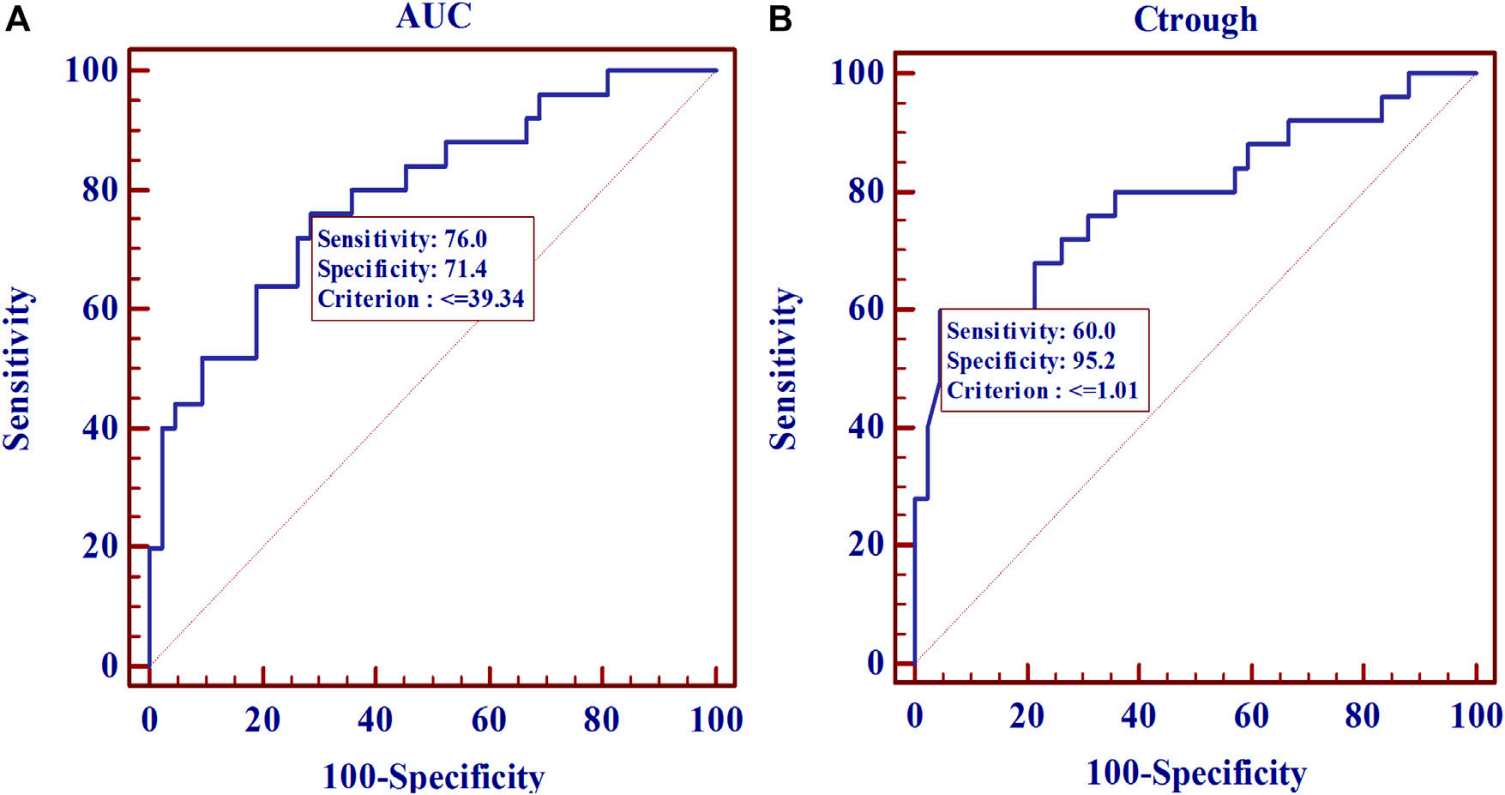
Receiver operating characteristic (ROC) curve estimates for the 67 SLE patients. **(A)**, for MPA AUC (AUC_ROC_: 0.790; *p* < 0.0001). (B), for C_trough_ (AUC_ROC_: 0.792; *p* < 0.0001).

From the ROC analysis, a threshold AUC value of 39 μg h/ml provided the best tradeoff between sensitivity (76%) and specificity (71%) (Figures 5A,B). For C_trough_, a threshold value of 1.01 μg/ml was associated with a sensitivity of 60% and a specificity of 95.2%. After AUC of 39 μg h/ml or C_trough_ of 1.01 μg/ml, the curve plateaued.

**FIGURE 6:**
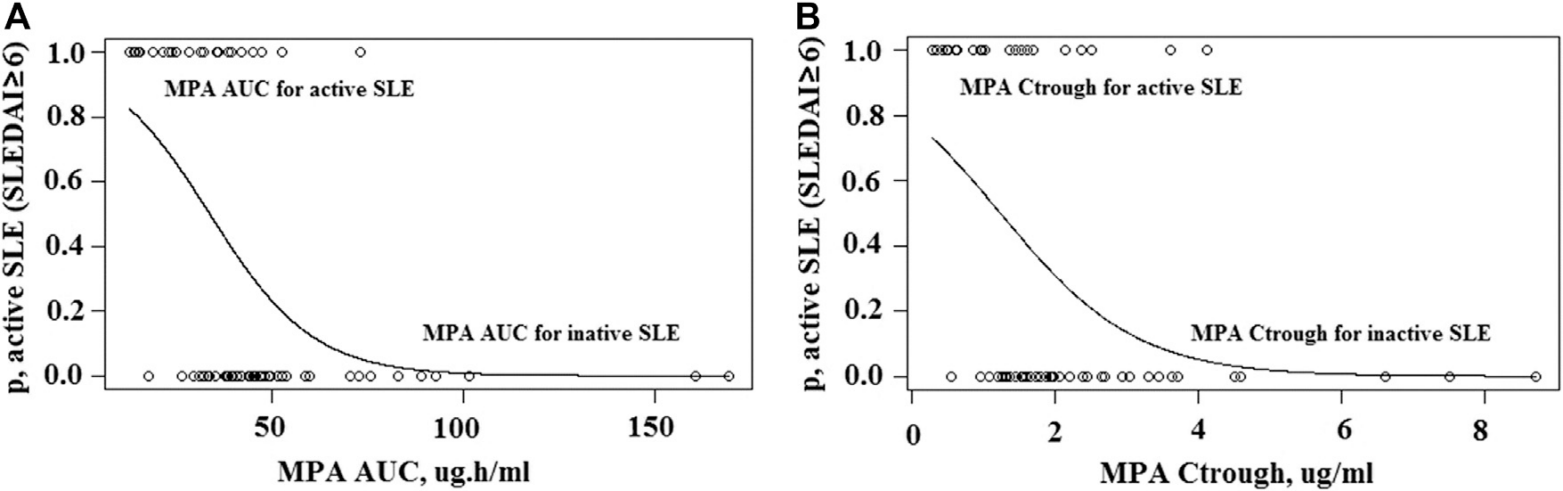
Two-parameter logistic regression. **(A)**, Sigmoid logistic regression relationship for MPA AUC and likelihood of active SLE. **(B)**, Sigmoid logistic regression relationship for MPA C_trough_ and likelihood of active SLE.

The 2-parameter logistic regression yielded a PK-pharmacodynamic (PD) relationship that was highly statistically significant (*p* < 0.0001). The logistic regression coefficients were 2.48 and −0.074 for *β*_0_ and *β*_1_, yielding the sigmoidal curve shown in Figure 6A. This curve illustrates the reduction in probability of active SLE as MPA AUC increases, with 50% of maximal efficacy occurring at an AUC of 34 μg h/ml (Table 3). The logistic regression with C_trough_ was also statistically significant (*p* < 0.0001). The logistic regression coefficients from the fit were 1.30 and −1.05 for *β*_0_ and *β*_1_, respectively (Figure 6B). This C_trough_ associated with 50% of maximal efficacy is 1.24 μg/ml (Table 3).

**TABLE 3:** MPA exposure attaining different probability of efficacy.

Probability of efficacy	Probability of active SLE (%)	AUC_0-12_ (μg.h/ml)	C_trough_ (μg/ml)
90% efficacy	10	63	3.33
80% efficacy	20	52	2.56
50% efficacy	50	34	1.24

a MPA AUC_0-12_ = mycophenolic acid area under the plasma concentration-time curve from 0 to 12 h.

**FIGURE 7:**
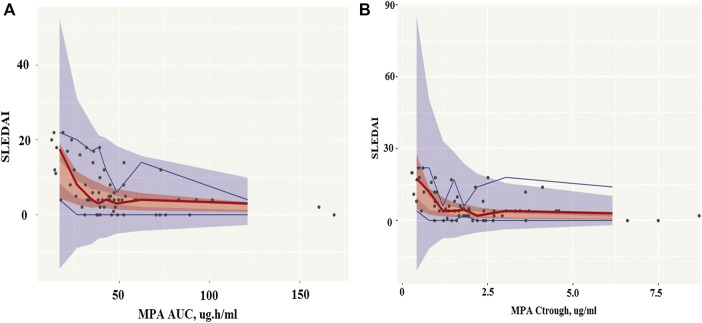
Visual predictive check (VPC) of mycophenolic acid models. **(A)**, The AUC-response model. **(B)**, The C_trough_-response model. Circles represent the observed blood concentrations; Lines represent the median, 5 and 95th percentiles of simulations (n = 1,000).

For exposure-response modeling, an inhibitory E_max_ model with AUC was established. Due to the lack of patients with severe SLE activity (SLEDAI score >22), E_0_ was fixed to 105. According to the theory, if the maximum effect is total inhibition of the baseline response, then E_max_ and E_0_ will have the same value. Therefore, E_max_ was also fixed to 105. There was no improvement when γ (hill coefficient) was included in the model. Parameter estimates of the MPA AUC-response model are summarized in Table 4. Bootstrap 95% CI_S_ for exposure-response model parameters were successfully estimated and all final parameter estimates were within those intervals, indicating a stable model (995 successful). Visual predictive check results also showed appropriate predictive performance of the model in that 95% CI of actual observations lay within the simulated 95% CI (Figure 7A). Besides, most of the observations are within 95% CI of actual observations. The result of the final model including AUC showed that the AUC should be 32 μg h/ml or higher to keep control SLE activity less than 6, and the AUC above 50 μg h/ml probably would result in disease control of SLE activity less than 4. Similarly, an E_max_ model of the relationship between C_trough_ and response described the data well. Parameter estimates of MPA C_trough_-response model are summarized in Table 4. Both bootstrap (991 successful) and visual predictive check supported the prediction of the C_trough_-response model (Figure 7B). The results indicated that a minimum MPA concentration of 1.1 ug/ml is needed (SLEDAI score <6) with concentration of 1.7 ug/ml associated with good reponse (SLEDAI score <4).

**TABLE 4:** Parameter estimates of the mycophenolic acid exposure-response model and bootstrap validation.

Parameter	Exposure-response model		Bootstrap n = 1,000		
	Population estimate	RSE (%)	Median	95% CI	
AUC-response model: E = E_0_-E_max_ ·AUC/(EC_50_+AUC)					
E_0_	105 (fixed)	—	—	—	
E_max_	105 (fixed)	—	—	—	
EC_50_ (ug·h/ml)	1.97	14.3	1.99	1.43–2.54	
Inter-individual variability					
EC_50_ (%CV)	31.6	49.4	30.6	13.7–49.5	
Residual error model					
Proportional (%CV)	76.7	18.5	76.3	63.6–91	
C_trough_-response model: E = E_0_-E_max_ ·C_trough_/(EC_50_+C_trough_)					
E_0_	105 (fixed)	—	—	—	
E_MAX_	105 (fixed)	—	—	—	
EC_50_ (ug/ml)	0.0664	14.1	0.0667	0.0485–0.0866	
Inter-individual variability					
EC_50_ (%CV)	52.1	40.2	50.9	28.2–73.3	
Residual error model					
Proportional (%CV)	76.2	20.3	75.8	60.9–91.5	

RSE, Relative standard error; CI, confidence interval; E_0_ is the baseline of SLEDAI; Emax is maximal SLEDAI; EC_50_ is the AUC required for 50% maximal SLEDAI.

## Discussion

In the present study, Bayesian estimation was employed to estimate MPA AUC_0-12_ in pediatric patients with SLE using the limited sampling strategy. To our knowledge, only two studies developed PK models for MPA when used in children with SLE. Sherwin *et al.* developed a six-compartment model including a gallbladder compartment for enterohepatic recycling and bile release time related to meal times, with first order absorption and a single series of transit compartments (Sherwin et al., 2012). Woillard JB et al. developed a double gamma absorption model from rich PK profiles which allowed the absorption profiles of MPA to be described very accurately (Woillard et al., 2014). Due to the limited sampling strategy, we have chosen the model developed by Woillard *et al.* to estimate MPA AUC_0-12_ in pediatric patients with SLE on the basis of four samples. We feel confident that this model can be used to estimate individual AUC_0-12_ with fairly good accuracy and therefore allows for better dose optimization in children with SLE.

This study explored the association between MPA exposure and disease activity. In this population of 67 pediatric patients with SLE, we observed that both MPA AUC_0-12_ and C_trough_ were related to disease activity as assessed by SLEDAI at the time of AUC determination. Previous studies also have found a correlation between MPA AUC_0-12_ and SLE activity. In adult patients, Zahr *et al.* demonstrated that MPA AUC_0-12_ was significantly lower in patients with active SLE than in those with inactive disease (Zahr et al., 2010). In a population of 36 children with SLE, Woillard *et al.* also observed that AUC_0-12_ were related to disease activity (Woillard et al., 2014). However, the association between C_trough_ and SLEDAI was unclear. Neumann *et al.* reported that in 12 patients with SLE and 26 patients with ANCA-associated vasculitis receiving MMF for remission maintenance therapy, higher MPA trough levels provided better protection from recurrence of active disease (Neumann et al., 2008). Controversially in the previously mentioned study undertaken by Woillard *et al.*, the logistic regression analysis revealed no association between C_trough_ and SLEDAI. In the current study, the data suggest that the association between C_trough_ and disease activity is not worse than for MPA AUC_0-12_.

Cattaneo *et al.* reported an impact of glucocorticoids on MPA exposure in kidney transplant recipients. Dose-normalized MPA AUC_0-12_ was lower during the first month (high doses of steroids) than at month six post-surgery (low maintenance dose of steroids) (Cattaneo et al., 2002). But neither the prednisolone AUC_0-12_ nor daily doses of prednisone were correlated with MPA PK parameters in the study by Zahr *et al.* (Zahr et al., 2008). The involvement of steroids in PK of MPA is still not conclusive. In the present study, we investigated the possible interactions between prednisolone and MPA by multiple linear regression analysis. Daily doses of prednisolone was found to be related with MPA PK parameters. MPA AUC_0-12_ was significantly lower in pediatric SLE patients with high doses of prednisolone. However, additional confounding factors such as daily dose of MMF and albumin concentration interfered with MPA exposure. Furthermore, since we could not withdraw corticosteroids in patients with SLE, we cannot determine whether there was a possible interaction between corticosteroids and MMF.

The therapeutic target for MPA AUC_0-12_ of 30–60 μg h/ml has been firmly established in MMF-treated renal transplant recipients. In patients with SLE, the data is limited. Several studies have shown that an MPA AUC_0-12_ of 30–60 μg h/ml (Alexander et al., 2014) or 45–60 μg h/ml (Zabotti et al., 2015) were correlated with a better outcome in adult SLE patients. In a population of 71 adult patients with SLE, Zahr *et al.* found MPA AUC_0-12_ was the only parameter associated with SLE activity and proposed a target AUC_0-12_ threshold of 35 μg h/ml (Zahr et al., 2010). In 19 pediatric patients with lupus nephritis or discoid lupus, Sagcal-Gironella *et al.* reported that an MPA AUC_0-12_ of 30 μg h/ml or higher was associated with improved disease control, whereas MPA AUC_0-12_ lower than that were not (Sagcal-Gironella et al., 2011). An MPA AUC_0-12_ > 45 μg h/ml was proposed to significantly associated with therapeutic response in childhood-onset lupus nephritis in the study by Godron-Dubrasquet *et al* (Godron-Dubrasquet et al., 2020). In the present study, an MPA AUC_0-12_ of 39 μg h/ml or a C_trough_ of 1.01 μg/ml was found as the target threshold for SLE in pediatric patients by ROC analysis, which is higher than the target MPA AUC_0-12_ thresholds proposed by Zahr *et al* (Zahr et al., 2010) and Sagcal-Gironella *et al* (Sagcal-Gironella et al., 2011), but lower than the AUC_0-12_ threshold reported by Godron-Dubrasquet *et al* (Godron-Dubrasquet et al., 2020). In general, the target MPA AUC_0-12_ threshold for SLE patients proposed by our team is consistent with that reports by other previous studies. This small discrepancy may be due to the different severity of disease activity of the included patients, different concentration measurement methods, and different statistical methods. A logistic regression model was used to represent the relationship between MPA AUC_0-12_ and likelihood of active SLE as measured by the SLEDAI. An MPA AUC_0-12_ of 34 μg h/ml was found to predict an effective control of SLE activity less than six in 50% of the patients, whereas 52 and 63 μg h/ml would yield effective control in 80% and 90 of the patients, respectively. Similarly, a C_trough_ of 1.24 mg/L would be expected to yield in 50% of the patients an effective control, whereas 2.56 and 3.33 mg/L are associated a SLE activity less than six in 80% and 90 of the patients, respectively. Although this finding will require further validation before it can be implemented clinically, an MPA AUC_0-12_ level of 34 μg h/ml and C_trough_ level of 1.24 mg/L or higher were associated with disease control of SLE activity less than 6, whereas MPA AUC_0-12_ or C_trough_ levels lower than that were not. In order to further explore the relationship between MPA exposure and disease activity, we herein first developed sigmoidal exposure-response models, which predict the probability of outcomes themselves rather than particular outcomes (0, 1) by logistic regression. The results were consistent with the logistic regression, and an MPA AUC_0-12_ of 32 μg h/ml and C_trough_ level of 1.1 mg/L or higher would keep effective control of SLE activity less than 6. An AUC_0-12_ above 50 μg h/ml or a C_trough_ above 1.7 ug/ml were identified to be associated with disease control of SLE activity less than 4. The accuracy and predictive performance of the final model were satisfactory, which implied that the use of AUC_0–12h_ or C_trough_ as an exposure variable was acceptable.

Although a fixed-dose regimen is still the current convention in MPA dosing, we observed a large variability of MPA PK/PD. Model-informed precison dosing appears particularly important in our SLE patients, and the target AUC_0-12_ and C_trough_ thresholds associated with active disease have been identified, although an upper limit cannot be defined because of the lack of toxicity in this cohort. Some limitations of this study should be considered. First, two forms of mycophenolate mofetil were used. As our study indicated, clinically relevant differences in PKs of these two formulation exist. However, the major findings of this study which show the exposure cut-offs associated with clinical outcomes, are not affected by formulations. Second, this is a retrospective cross-sectional study. Prospective longitudinal studies are clearly needed to propose a possible therapeutic target range for MPA AUC_0-12_ or trough concentrations. Yet, the results from this study presented herein may be seen as a valuable addition to the current knowledge of MMF PK in SLE. In addition, it would be preferred to characterize MPA PK using free drug concentrations. As albumin concentration in patients with active SLE may be subnormal, this factor will affect drug exposure. Lastly, MPA concentrations were measured by an immunoassay which is commonly used in clinical labs. Due to possible cross-reactions, it may give higher results compared to other assays such as high performance liquid chromatography (HPLC) test. However, in areas/countries of low-resource, HPLC and LC-MS/MS technologies may not be readily available in the clinical settings. Therefore, our results may provide “real-world” values to guide TDM of MMF in patients with SLE.

## Conclusion

A published population PK model was successfully used as prior information to estimate MPA AUC_0-12_ in our pediatric patients with SLE. Both AUC_0-12_ and C_trough_ were correlated with SLE activity. An AUC_0-12_ above 50 μg h/ml or a C_trough_ above 1.7 ug/ml were recommended to provide a good clinic improvement.

## Data Availability Statement

The original contributions presented in the study are included in the article/Supplementary Material, further inquiries can be directed to the corresponding author.

## Ethics Statement

The studies involving human participants were reviewed and approved by Fudan Children’s ethics committee. Written informed consent to participate in this study was provided by the participants' legal guardian/next of kin.

## Author Contributions

YC performed the research, analyzed data and wrote the manuscript; ZL designed the research; LS and YL collected data; MD and TM gave good suggestions and revised the manuscript; HX, AV, and HB provided valuable edits and comments.

## Conflict of Interest

The authors declare that the research was conducted in the absence of any commercial or financial relationships that could be construed as a potential conflict of interest.
