# Supplementary material for: PK/PD Study of Mycophenolate Mofetil in Children With Systemic Lupus Erythematosus to Inform Model-Based Precision Dosing
Source: Front Pharmacol. 2020 Dec 21;11:605060. doi: 10.3389/fphar.2020.605060 (PMC7819284; doi:10.3389/fphar.2020.605060)
Supplement: Supplementary file 1 [file datasheet1.docx]

**Supplementary material**

**Model evaluation**

**Methods**

The predictive performance of the model was evaluated graphically, and by means of precision and bias. The mean prediction error (MPE) and mean absolute prediction error (MAE) were used as measures of precision and bias [1], respectively. They are calculated by the following equations:

 (S1)

 (S2)

where OBS_i_ represents the observed concentration of the ith subject, PRED_i_ represents the individual predicted concentration of the ith subject. In addition, the percentage of patients with MPE within ±20% (F_20_) or within ±30% (F_30_) was calculated. High percentage of prediction errors within ±20% or ±30% was considered acceptable.

**Results**

**Model evaluation**

A comparison of the population model predicted and post hoc Bayesian-predicted MPA serum concentration-time profiles and the observed concentrations for four typical subjects are shown in Supplementary *Figure-S1*. Regression analysis between observed and Bayesian individualized predicted concentrations also shows that the model can predict well (Supplementary *Figure-S2*). The precision and bias also indicated that the model provided good prediction (Supplementary *Table-S1*). We also compared the populations in SLE model published by Woillard and our study. The population parameters in Woillard and Individual parameters are close (Supplementary *Table-S2*). Besides, the patients’ characteristics of children in both studies are similar (Supplementary *Table-S3*).

References

[1] van der Meer AF, Marcus MA, Touw DJ, Proost JH, Neef C. Optimal sampling strategy development methodology using maximum a posteriori Bayesian estimation. Ther Drug Monit 2011;33:133-46.

**SUPPLEMENTARY FIGURE LEGENDS**

*Supplementary* *Figure-S1.* Four typical examples of fitted PK profiles (Circles are observed concentrations while solid line represent the Posthoc Bayesian predicted concentration-time profile and dash line represent the model-based predicted concentration-time profile)

*Supplementary Figure-S2.* Regression analysis between observed and Bayesian individualized predicted concentrations (a, predose; b, 20 Min; c, 1h; d, 3h)

*Supplementary Table-S1* Precision and bias of the population PK model

| Concentrations | MPE% | MAE% | F_20_ | F_30_ |
| --- | --- | --- | --- | --- |
| **C_0_** | 0 | 6% | 94% | 96% |
| **C_20 min_** | 3% | 3% | 94% | 97% |
| **C_1h_** | 4% | 5% | 96% | 96% |
| **C_3h_** | 3% | 3% | 96% | 97% |

*Supplementary Table-S2* Population PK parameter estimates of MPA in pediatric patients with SLE

| Parameter | Population estimates in Woillard JB study | Individual estimates in our validation study | Range of individual estimates in our validation study (Range) |
| --- | --- | --- | --- |
| CL (L/h) | 19.2±13.2 | 26.7±10.4 | 5.0-54.2* |
| V (L) | 24.8±13.5 | 19.5±8.0 | 4.5-50.8* |
| FR | 0.3±0.2 | 0.4±0.2 | 0.08-0.8 |
| K | 49.9±14.1 | 53.7±6.0 | 41.1-64.7 |
| K_2_ | 27.6±10.7 | 27.2±4.7 | 16.0-50.0 |
| N | 20.2±6.57 | 19.2±3.0 | 15.5-28.3 |
| N_2_ | 29.2±12.09 | 31.5±5.7 | 15.7-56.8 |

*Except where indicated otherwise, values are the mean±SD.

*CL, clearance; V, volume of distribution; FR, fraction of drug absorbed through the faster route; K and K_2_ , fractional absorption rate; N and N_2_, number of transit compartments

*Allometrically scaled CL and V with body weight

*Assay error (mg/L): $0.226+0.0039*C+0.0033*C^{2}-0.00005*C^{3}$

*Supplementary Table-S3* Characteristics of children in Woillard JB and our validation study

|  | Woillard JB study (n = 36) | Our validation study (n = 67) |
| --- | --- | --- |
| Girls, no. (%) | 26 (72%) | 56 (84%) |
| Age, year | 12.9±2.6 (4.7-16.7) | 13.2±2.8 (4-18) |
| Weight, kg | 45.8±16.1 (16.0-96.0) | 45.0±11.1 (18-73) |
| Albumin, g/l | 36.9±4.1 (29.0-44.0) | 38.6±6.4 (21.9-47.7) |
| MMF dosage, mg | 728±255 (300-1250) | 1039±296 (500-2000) |
| SLEDAI score | 6±6 (0-20) | 7±6 (0-22) |
| Patients having an active disease at the time of sampling (SLEDAI ≥6) n (%) | 16 (44.4%) | 25 (37.3%) |
